# Can Functional Traits Explain Recent Changes in Abundance of Alpine Plant Species?

**DOI:** 10.1002/ece3.73806

**Published:** 2026-06-07

**Authors:** Feline Peters, Jana Weghorst, Mariana Paetzolt, Andrea Lamprecht, Harald Pauli, Manuela Winkler, Patrick Saccone, Peter Hietz

**Affiliations:** ^1^ Institute of Botany BOKU University Vienna Vienna Austria; ^2^ GLORIA Co‐Ordination, Institute for Interdisciplinary Mountain Research, Austrian Academy of Sciences & Institute of Botany BOKU University Vienna Austria; ^3^ Institute of Botany of the Czech Academy of Sciences Průhonice Czech Republic

**Keywords:** alpine plants, climate change, ecophysiology, functional trait, long‐term monitoring, species abundance, species frequency

## Abstract

Climate change is driving significant changes in alpine ecosystems, where temperature‐sensitive plant communities are particularly vulnerable. As plants migrate to higher elevations, understanding the mechanisms behind these changes is critical for predicting ecosystem dynamics. Morphological, physiological, and chemical functional traits may help understand species' responses to environmental change. This study examines whether functional traits can explain recent changes in presence and cover of alpine plant species in permanent plots. We analyzed vegetation data from repeated surveys of permanent plots at two sites. Morphological traits (e.g., plant height, leaf area) and physiological traits (e.g., frost and drought resistance) were measured locally for common species and supplemented with data from the TRY trait database to broaden species coverage. Linear models were applied to assess the relationships between traits and abundance changes (presence and cover), using multiple metrics. Our results reveal that plant height and leaf area are significant predictors of species abundance changes, emphasizing the role of morphological traits in shaping alpine plant communities. In contrast, physiological traits showed limited explanatory power. Notably, leaf carbon content emerged as a key predictor, suggesting that conservative strategies may provide advantages under warming conditions. Measures describing biomass dynamics (e.g., cover) differed from those describing establishment (e.g., presence), highlighting the multifaceted nature of species responses to environmental changes. Although our findings emphasize the importance of competitive interactions and resource acquisition, the study is limited by the absence of traits related to heat tolerance and prolonged warming. Future research should address these gaps to better understand the impacts of sustained temperature increases on alpine ecosystems.

## Graphical abstract

We tested whether plant functional traits can explain recent changes in alpine species abundance using long‐term monitoring data from Austria. Morphological traits like plant height and leaf area predicted species changes, while physiological stress‐tolerance traits showed no significant relationships. Our results suggest competitive ability matters more than stress tolerance for understanding alpine vegetation responses to climate warming.

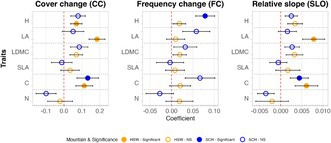

## 1 Introduction

Climate change is one of the most pressing environmental challenges of our time, with particularly profound implications for biodiversity in temperature‐limited ecosystems where it is reshaping community composition and species distributions (Lamprecht et al. 2018; Steinbauer et al. 2020). Central European mountains, including the Alps, have warmed at roughly twice the global rate—approximately 0.3°C± 0.2°C per decade (IPCC 2018; Pepin et al. 2015)—amounting to about 2°C since 1900 in the Alps, with further warming projected by the end of the 21st century depending on emission scenario (Kotlarski et al. 2023). This warming is accompanied by shifts in precipitation patterns, reductions in snow cover duration, and upward shifts in the 0°C isotherm (Kotlarski et al. 2023), all with direct consequences for alpine plant communities. Due to the compression of thermal life zones, the pronounced topography and the orographic isolation, alpine areas are biodiversity hotspots rich in endemic species (Körner 2021). While mountains cover approximately 35% of Europe's land area (European Environment Agency 2010), only 2% lies above the treeline (Testolin et al. 2020). Yet this alpine zone hosts approximately 20% of the continent's native vascular plants (Väre et al. 2003). These features also make alpine plant communities very sensitive to climate shifts, and multiple vegetation changes have already been documented across European summits, including thermophilization (Lamprecht et al. 2018), increasing species richness (Pauli et al. 2012; Steinbauer et al. 2018), and shrub expansion (Dullinger et al. 2003; Lamprecht et al. 2021; Vanneste et al. 2026). Because these rapid environmental changes occur over small spatial scales, alpine ecosystems are particularly well suited for studying plant community responses to climate change (Grabherr et al. 2010; Körner 2021).

While these vegetation changes are well documented, the underlying mechanisms driving vegetation changes remain less clear. Predicting how climate change will reshape community composition and ecosystem functioning therefore depends on identifying which functional traits allow species to persist, or are responsible for their increase or decline. Plant functional traits are defined as “any morphological, physiological, or phenological feature measurable at the individual level, from the cell to the whole plant” (Violle et al. 2007). These traits fall into categories that capture different trade‐offs in how plants grow, survive, and cope with their environment (Díaz et al. 2016). *Morphological* traits relate to light capture, competitive ability, and resource acquisition; *physiological* traits determine tolerance to abiotic stresses such as frost and drought; and *chemical* traits reflect nutrient economics and structural investment (Pérez‐Harguindeguy et al. 2013; Wright et al. 2004). Together, these traits reflect fundamental ecological strategies related to growth, survival, and reproduction, providing a mechanistic link between environmental conditions and species performance (Lavorel and Garnier 2002; Pérez‐Harguindeguy et al. 2013; Tomlinson 2025).

While morphological traits, such as plant height and specific leaf area (SLA), have been shown to predict species responses to temperature shifts (Bjorkman et al. 2018; Henn et al. 2024; Soudzilovskaia et al. 2013), physiological traits remain underrepresented in trait‐based studies of alpine vegetation changes. This is likely because they are more complex and time‐consuming to measure, yet they capture plant tolerance to stress factors such as frost and drought more directly than morphological traits (Larcher 2003; Neuner 2014). This distinction matters in alpine environments, where ongoing warming is expected to reduce cold stress and potentially intensify drought‐related stress, which can favor species with traits conferring efficient water use or greater drought tolerance (Soudzilovskaia et al. 2013; Visakorpi et al. 2023). Studies that do incorporate ecophysiological traits have typically analyzed them within multivariate trait spaces rather than isolating the effects of individual traits on species performance (Henn et al. 2024; Visakorpi et al. 2023). Others rely on short‐term manipulative experiments which capture immediate responses to discrete events, but cannot reflect the cumulative dynamic response of communities to ongoing change (Brancaleoni et al. 2025; Madsen‐Hepp et al. 2023). Our study addresses these gaps by examining individual ecophysiological traits related to frost and drought resistance alongside morphological and chemical traits, relating them to long‐term observational species abundance data from alpine to subnival ecosystems.

Beyond the choice of traits, the methodology used to quantify species responses can also influence ecological conclusions. Species abundance can be measured using different metrics, such as frequency (derived from presence/absence records across sampling units) and cover (proportion of ground area occupied by a species), that capture distinct ecological dimensions, and the choice between them may influence trait‐based inferences. Frequency reflects spatial distribution patterns and colonization/extinction dynamics across sampling locations, making it particularly sensitive for detecting newly established or rare species (Helm et al. 2024), which can contribute substantially to functional diversity of the community (Mouillot et al. 2013). In contrast, cover reflects biomass dynamics and competitive performance within established plant communities, better depicting dominance and resource utilization patterns (Chiarucci et al. 1999; Moradi et al. 2025). These species response metrics thus provide complementary views of community composition and traits that explain them may differ accordingly. Stress tolerance traits may primarily determine whether a species establishes or disappears from a site, while traits associated with growth rate and resource acquisition may govern whether it gains or loses biomass within an existing community (Lisner and Lepš 2020). Yet, few studies have examined whether trait‐environment relationships differ depending on the metric used.

This study explores how morphological, physiological, and chemical traits influence the frequency and cover changes of alpine plant species in long‐term monitoring sites in the Austrian Alps. Using monitoring data spanning nearly three decades, we compare how functional traits relate to shifts in species frequency versus cover, testing whether these species' response metrics capture different ecological dimensions of climate‐driven change. We hypothesize that (Albert 2015) physiological traits, which reflect plant adaptations to frost and drought stress, will better explain observed occurrence and cover changes than morphological traits (such as plant height and leaf traits); and (Alexander et al. 2015) different functional traits will account for changes in cover (biomass accumulation or loss) versus frequency (colonization or disappearance), as these represent distinct ecological processes that may be governed by different physiological constraints.

## 2 Methods

### 2.1 Study Site and Vegetation Surveys

This study was conducted at two sites of the Global Observation Research Initiative in Alpine Environments (GLORIA, www.gloria.ac.at; Pauli et al. 2015): Schrankogel (SCH), located in the Stubaier Alpen in the central Tyrolean Alps (47.241°N, 11.557°E, summit at 3497 m a.s.l.), and Hochschwab (HSW), located in the northeastern Alps (47.375°N, 15.8322°E, summit at 2277 m a.s.l.). At Schrankogel, the bedrock is composed of siliceous substrate, predominantly gneiss (Geological Survey of Austria 2022), with permanent plots spanning the alpine‐(sub)nival ecotone (2911–3457 m), and with vegetation ranging from closed *Carex curvula* grasslands, to open, scattered plant assemblages of the *Androsacion alpinae* on scree and rock (Lamprecht et al. 2018). At Hochschwab, the bedrock is composed of calcareous substrates, predominantly limestone (Poggio et al. 2021), with plots covering a small elevational range between 1919 m, where *Pinus mugo* shrubs transition into (sub)alpine grasslands to 2277 m, with *Caricetum firmae* grasslands with snowbed communities and scree vegetation (Dullinger et al. 2003).

The Schrankogel summit comprises four subsites: on the SW‐, S‐, SE‐ and E slope (hereafter referred to as ‘blocks’). Over 1000 1‐m^2^ plots were established across these blocks, organized into 21 transects ranging from 1 to 30 m in length and 1–3 m in width (for details see Lamprecht et al. 2018). The study site was established and first surveyed in 1994; re‐surveys were conducted in 2004, 2014, and 2023. Hochschwab, following the Multi‐summit approach standard for GLORIA sites (Pauli et al. 2015), contains 4 summits. Each summit contains four 1‐m^2^ plots in each cardinal direction, totaling 64 plots on Hochschwab. The site was established and first surveyed in 2001; re‐surveys were conducted in 2008, 2015, and 2022. At both study sites, the percentage cover of each vascular plant species was visually estimated in each 1 m^2^ plot. Together, these two study sites encompass a broad range of alpine environments in the Austrian Alps, from the lower alpine zone on calcareous bedrock at Hochschwab to the upper alpine and nival zones on siliceous bedrock at Schrankogel, thereby capturing contrasting community compositions, species pools, and environmental conditions.

### 2.2 Plant Sampling for Trait Measurements

Plant sampling for trait measurements was conducted at Schrankogel in 2021 and 2023, and at Hochschwab in 2022. Plants were sampled near the permanent plots on both mountains: directly surrounding the plots on Schrankogel, and approximately 10 m below the GLORIA plots on Hochschwab to avoid disturbance. We prioritized abundant species as recorded in the plots but also included less abundant species to represent different growth forms and lineages. We sampled 33 species at Schrankogel and 48 species at Hochschwab representing > 70% of the total vascular plant cover at each site (see Tables S2 and S3 for species lists). For each species, five mature and healthy individuals were sampled at a single location, with at least 1 m between individuals to avoid collecting multiple ramets from a clone. For the analysis of frost tolerance, additional leaves were pooled from neighboring individuals to obtain sufficient material. Leaves were wrapped in wet tissue paper and stored in plastic bags during fieldwork and transport to the laboratory. Leaves were rehydrated at 4°C overnight to ensure full saturation prior to all measurements, which were started within 24 h after sampling.

### 2.3 Trait Measurements

We measured traits that describe plant morphology, physiology, and leaf chemistry. Morphological traits capture strategies related to light capture, competitive ability, and resource acquisition (Pérez‐Harguindeguy et al. 2013), and included leaf area (LA), SLA, leaf dry matter content (LDMC), leaf thickness (LT), leaf aspect ratio (AR), leaf shape complexity (solidity), and vegetative plant height (H). Physiological traits directly measure plant stress tolerance to frost and drought, reflecting functional acclimation to alpine environments (Larcher 2003; Neuner 2014), and included chlorophyll content (Chl), osmotic concentration of saturated leaves (Osm), leaf frost tolerance (LT50), minimum leaf conductance (gmin), and leaf succulence (Suc). Chemical traits, which reflect nutrient economics and structural investment (Ma et al. 2018; Wright et al. 2004), included leaf carbon content (C) and leaf nitrogen content (N); these were measured locally for species at Schrankogel but not at Hochschwab. The ecological significance and methodological source of the traits measured are summarized in Table 1; details of our measurement protocols are provided in the Supporting Information.

**TABLE 1:** Functional traits analyzed in this study. For each trait, the table provides its abbreviation, definition, unit of measurement, ecological significance, and supporting reference(s).

Functional trait	Explanation	Unit	Ecological significance	References
AR	Aspect ratio	Leaf length/leaf width	Light capture, climate adaptation	Li et al. (2020)
C	Leaf carbon content	% dry weight	Structural investment, leaf construction cost	Ma et al. (2018)
Chl	Chlorophyll content	Relative SPAD units	Photosynthetic capacity	Mielke et al. (2010)
*g*_min_	Minimum conductance	g cm^−2^ h^−1^	Drought resistance	Cape and Percy (1996); Liang and Ye (2024)
H	Plant vegetative height	cm	Light capture and competitiveness	Moles et al. (2009)
LA	Leaf area	cm^2^	Light capture, gas exchange, thermal regulation	Montès et al. (2024)
LDMC	Leaf dry matter content	g dry weight g^−1^ fresh weight	Mechanical stability, stress resistance	Pérez‐Harguindeguy et al. (2013); Vile et al. (2005)
LT	Leaf thickness	mm	Resource acquisition, stress resistance	Vile et al. (2005)
LT_50_	Temperature when yield (*F*_v_/*F*_m_) decreases by 50%	°C	Frost resistance	Neuner et al. (2020); Taschler and Neuner (2004)
N	Leaf nitrogen content	% dry weight	Photosynthetic capacity, nutrient economics	Wright et al. (2004)
Osm	Osmotic concentration	mmol kg^−1^	Drought and frost resistance	Bartlett, Scoffoni, Ardy, et al. (2012); Bartlett, Scoffoni, and Sack (2012)
SLA	Specific leaf area	mm^2^ mg^−1^ dry weight	Photosynthetic capacity, light capture, water loss and mechanical stability	Pérez‐Harguindeguy et al. (2013); Reich et al. (1998)
Solidity	Shape complexity of a leaf	Area of particle divided by its convex hull	Light capture, water loss and thermal regulation	Montès et al. (2024)
Suc	Succulence, amount of water a leaf can hold	g cm^−2^	Water storage capacity	Vendramini et al. (2002)

### 2.4 Data Compilation and Analysis

During data quality control, visual inspection of trait distributions per species identified no outliers. The remaining trait values were averaged across the five replicates per species and site to obtain a species × trait matrix for each site. To increase species coverage, we supplemented our locally measured data with trait values from the TRY database (Kattge et al. 2020; TRY data request 21179). TRY data were available for commonly measured traits (height, leaf area, SLA, leaf dry matter content, leaf carbon content, leaf nitrogen content), but not for the physiological traits such as minimum leaf conductance, leaf freezing tolerance, and osmotic concentration, which therefore remained available only from the local dataset. This produced two parallel datasets for each site: a local‐only dataset (excluding leaf carbon and nitrogen content due to incomplete local coverage) and a combined local‐and‐TRY dataset. In total, we compiled trait values for up to 73 species at Schrankogel (33 locally sampled) and 143 species at Hochschwab (48 locally sampled). Not all traits were available for every species, and coverage varied both across traits and between locally measured and TRY‐supplemented data (Tables 3 and 4, Table S1).

Height, leaf area, SLA, aspect ratio, and succulence were log‐transformed to improve normality. All traits were then centered and scaled to unit variance prior to analysis. Since the two study sites differed in sampling design (blocks and transects on Schrankogel vs. four distinct summits on Hochschwab), geology (siliceous vs. calcareous bedrock), and elevation, we analyzed them separately. For each site, we ran two sets of analyses: one using locally collected trait data only, and one combining locally collected and TRY data. Models were fitted using all species for which data on the respective trait were available, resulting in variable trait‐specific sample sizes (Tables 3 and 4, Table S1). For the principal component analysis, which requires a complete species × trait matrix without gaps, missing values were imputed using MissForest (Stekhoven and Bühlmann 2012); missing TRY values were not imputed.

We quantified the change in species abundance in three ways: for both percentage cover and frequency, we calculated the relative change between the first and last survey. For these two measures, species cover and frequency were aggregated across all plots within each site, yielding a single value per species per survey (e.g., Rosbakh et al. 2025). This provides a straightforward summary of overall species trends at the site level but does not account for the nested sampling design (e.g., Lamprecht et al. 2018) and ignores surveys between the first and the last one. Because our monitoring data span nearly three decades with multiple surveys, we additionally fitted mixed‐effect models that include all survey years, accounting for the data structure and permit testing for statistical significance of changes. We used the relative changes in occurrence and cover, so that proportionally equivalent changes are treated equally and a change in cover from 1% to 2% has the same effect as a change from 10% to 20%. Thereby, the trait‐response relationship reflects patterns across all species and is not driven by a few dominant ones. We thus calculated (Albert 2015) relative cover change (Alexander et al. 2015) relative frequency change, and (Bartlett, Scoffoni, Ardy, et al. 2012) relative slopes from linear mixed‐effect models:

1. The relative cover change per species and site was calculated with the sum of the cover across all plots during the first and last survey as CC = $\frac{\text{cover last survey}-\text{cover first survey}}{\text{cover first survey}+\text{cover last survey}}$.
2. The relative change in frequency (i.e., the proportion of plots a species was found in) was calculated as the number of plots where a species was present within each site at the first and last survey as FC = $\frac{\text{frequency last survey}-\text{frequency first survey}}{\text{frequency first survey}+\text{frequency last survey}}$
3. For the last measure, we applied linear mixed effect (LME) models using species abundance data from all survey years at both sites. Rather than relying solely on first and last survey comparisons, which cannot detect unimodal trends or assess statistical significance, or testing each survey year individually, we calculated regressions of cover against survey year while accounting for the specific sampling designs at both study sites with mixed effect models using lme4 (Bates et al. 2015). These models predict species cover at the 1 m^2^ plot level with “year” as a fixed factor and include the nested sampling design on Schrankogel (plot/transect/block) and Hochschwab (plot/aspect/summit) as random effects. In contrast to frequency and cover change, this permits testing for the significance of observed changes for each species. To quantify relative changes in abundance for each species, we used the relative slope (SLO) of these models, defined as the regression slope divided by the average cover per species in all four survey years.

We performed principal component analyses (PCA) using the R package *factoextra* (Kassambara and Mundt 2020) to identify major trait axes representing the dominant dimensions of functional variation between species. Using only locally collected trait data, we extracted the first two principal components (PC1 and PC2, respectively) to quantify coordination in trait space. In addition to individual traits, these PCs, which can capture key ecological strategies and trade‐offs, were then used as predictors in linear models against the different measures of species abundance change. To test if plant functional traits influence the species' responses, we fitted simple linear models for each combination of species response metric (CC, FC and SLO) and predictor, with individual plant functional traits and PCs as predictors. To reduce the influence of random fluctuations in rare species, species were weighted by the square root of their cover in these regressions. We applied this approach to both our locally collected trait data and the combined local and TRY dataset, allowing us to assess trait effects across different scales of data availability. R v.4.1.3 (R Core Team 2025) was used for all statistical analysis.

## 3 Results

### 3.1 Species Abundance Changes

Schrankogel and Hochschwab showed contrasting changes in species abundance (Table 2). On Schrankogel, most of the species increased in frequency and cover at both site and plot levels, while on Hochschwab, species changes are more balanced across all three metrics, with similar numbers of species increasing and decreasing.

**TABLE 2:** Percentage of species showing increasing or decreasing trends at Schrankogel (SCH) and Hochschwab (HSW) based on three species response metrics: Relative cover change (CC), relative frequency change (FC), and relative slope (SLO). Values in brackets next to relative slope percentages represent the number of statistically significant changes. Where rows do not sum to 100%, the remaining species showed no change.

Study site	Number of species (*n*)	Species response metric	Increases (%)	Decreases (%)
Schrankogel	73	Relative cover change (CC)	69.9	30.1
		Relative frequency change (FC)	83.6	12.3
		Relative slope (SLO)	68.5 (20.5)	31.5 (17.8)
Hochschwab	143	Relative cover change (CC)	55.2	44.8
		Relative frequency change (FC)	33.6	46.9
		Relative slope (SLO)	54.5 (10.5)	45.5 (12.6)

### 3.2 Trait Ordination

**FIGURE 1:**
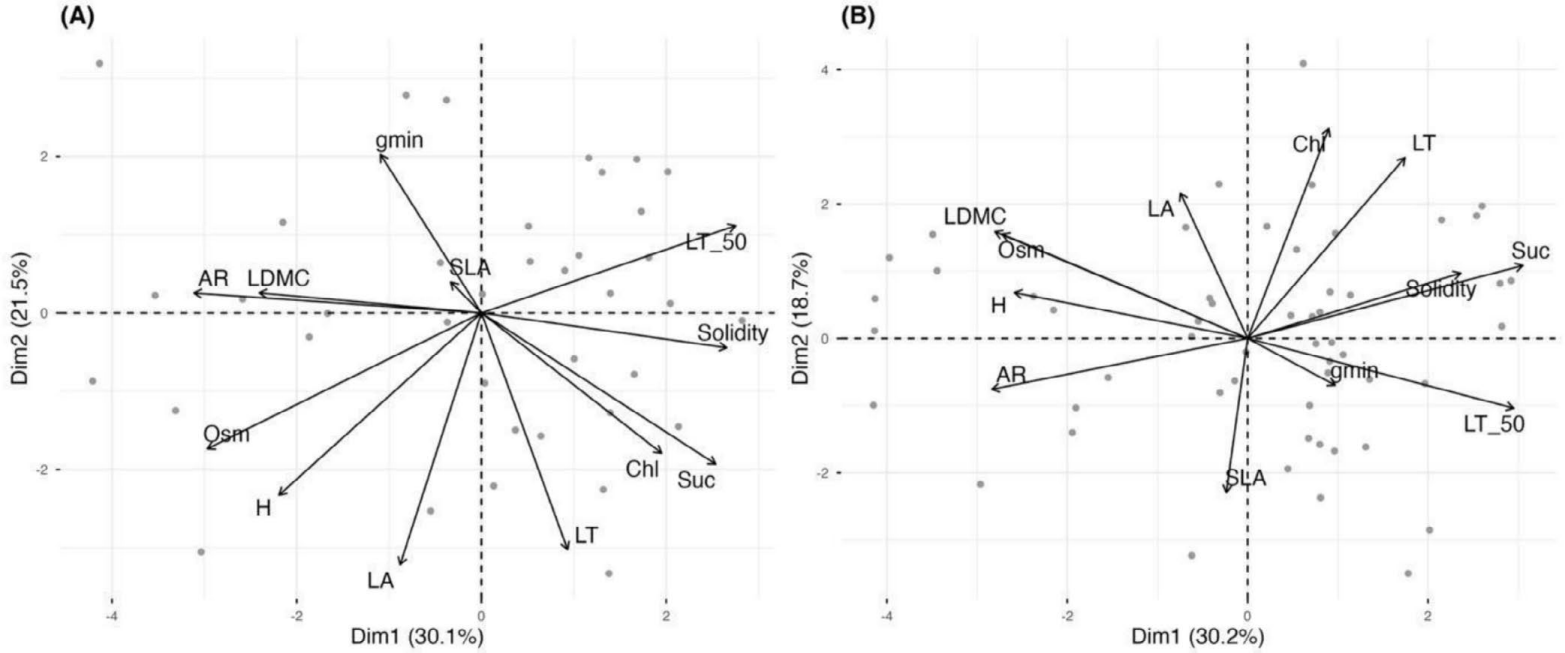
Principal component analysis of 12 leaf functional traits of alpine plants at Schrankogel (A) and Hochschwab (B). Each point represents a species‐level mean. Arrows indicate trait loadings on PC1 and PC2 (see Tables S2 and S3 for loadings and S4 for trait values).

Principal component analyses revealed similar levels of overall trait variation at the two sites, with the first two axes explaining around half of the variance in each case. The traits structuring this variation, however, differed between sites. At Schrankogel, PC1 explained 30.1% and PC2 21.4% of trait variation (Figure 1A). Aspect ratio and leaf dry matter content loaded negatively on PC1, while leaf freezing tolerance and solidity loaded positively. Leaf area, leaf thickness, and minimum leaf conductance scaled with PC2. At Hochschwab, 30.2% of trait variation is explained by PC1 and 18.7% by PC2 (Figure 1B). Leaf dry matter content and osmotic concentration loaded negatively on PC1, with leaf freezing tolerance and minimum leaf conductance loading positively. SLA scaled with PC2.

### 3.3 Are Traits Related to Measures of Change?

**FIGURE 2:**
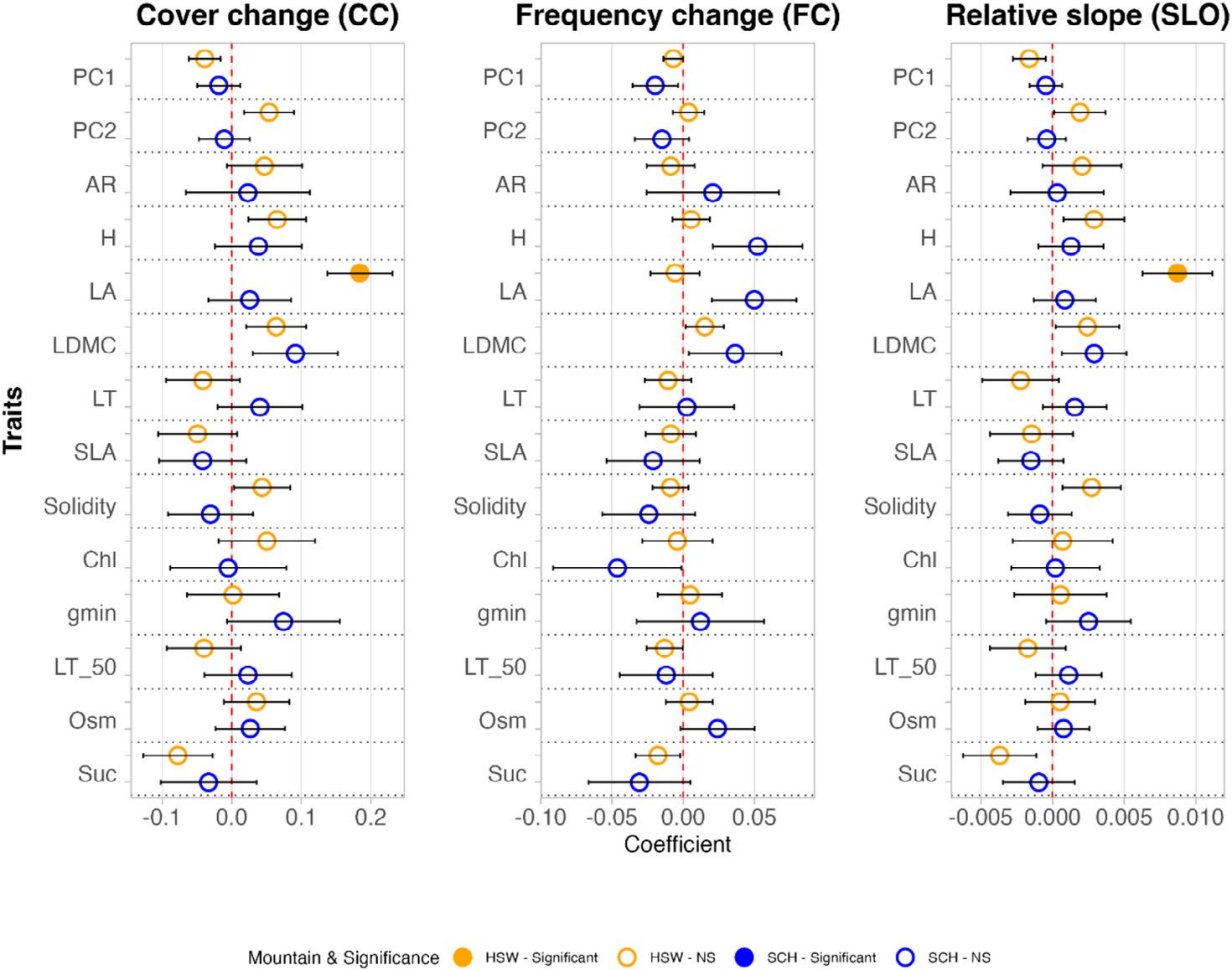
Coefficients from linear models with measure of species change and traits measured at the study sites. Traits are grouped into PCA‐derived (PC1, PC2), morphological, and physiological traits, separated by solid horizontal lines. Coefficients represent the change in relative cover change per scaled unit increase in each functional trait. Error bars show standard errors, significant relationships (*p* < 0.05) are indicated by full symbols, non‐significant relationships by empty symbols with blue representing Schrankogel (SCH) and orange Hochschwab (HWS). Number of observations, *R* and *p*‐values of the regressions are shown in Table 4 (SCH) and 5 (HSW).

Analysis of locally collected trait data revealed generally weak relationships between traits and species response metrics, with similar patterns at both sites (Figure 2, Table 3). The only significant associations were found at Hochschwab, where species with larger leaf area showed greater increases in cover and relative slope (CC: *p* < 0.001, *R* ^2^ = 0.30, SLO: *p* = 0.001, *R* ^2^ = 0.26).

**TABLE 3:** Summary of linear model performance for trait–abundance change relationships at two mountain sites (Hochschwab: HSW, Schrankogel: SCH), based on local trait data. *R* ^2^ and *p*‐values are shown for three measures of abundance change: Cover change (CC), frequency change (FC), and relative slope (SLO). N indicates the number of species per trait and site.

Trait	Mount	*N*_Obs	Cover change		Frequency change		Relative slope	
			*R* ^2^	*p*	*R* ^2^	*p*	*R* ^2^	*p*
PC1	HSW	44	0.063	0.099	0.023	0.326	0.045	0.165
	SCH	33	0.011	0.554	0.047	0.227	0.005	0.683
PC2	HSW	44	0.051	0.142	0.003	0.733	0.026	0.292
	SCH	33	0.003	0.772	0.02	0.438	0.003	0.763
AR	HSW	39	0.02	0.39	0.007	0.604	0.015	0.46
	SCH	33	0.002	0.796	0.006	0.659	0	0.92
H	HSW	39	0.063	0.124	0.005	0.676	0.048	0.181
	SCH	33	0.012	0.544	0.082	0.106	0.01	0.575
LA	HSW	39	0.295	0	0.003	0.74	0.257	0.001
	SCH	33	0.006	0.666	0.083	0.103	0.005	0.694
LDMC	HSW	39	0.056	0.147	0.033	0.266	0.032	0.279
	SCH	33	0.067	0.146	0.039	0.27	0.051	0.205
LT	HSW	38	0.017	0.439	0.012	0.517	0.019	0.412
	SCH	29	0.016	0.512	0	0.939	0.018	0.494
SLA	HSW	39	0.02	0.392	0.007	0.62	0.007	0.617
	SCH	33	0.014	0.509	0.013	0.523	0.014	0.512
Solidity	HSW	39	0.031	0.288	0.014	0.481	0.046	0.189
	SCH	24	0.011	0.621	0.024	0.466	0.007	0.693
Chl	HSW	33	0.017	0.472	0.001	0.869	0.001	0.84
	SCH	20	0	0.952	0.055	0.318	0	0.949
*g*_min_	HSW	31	0	0.977	0.002	0.836	0.001	0.865
	SCH	23	0.038	0.37	0.003	0.789	0.033	0.405
LT_50_	HSW	36	0.016	0.457	0.03	0.31	0.012	0.519
	SCH	28	0.005	0.713	0.005	0.718	0.009	0.628
Osm	HSW	33	0.018	0.455	0.002	0.799	0.001	0.831
	SCH	32	0.009	0.598	0.028	0.362	0.006	0.673
Suc	HSW	39	0.061	0.13	0.034	0.263	0.054	0.156
	SCH	33	0.007	0.633	0.023	0.395	0.005	0.706

**FIGURE 3:**
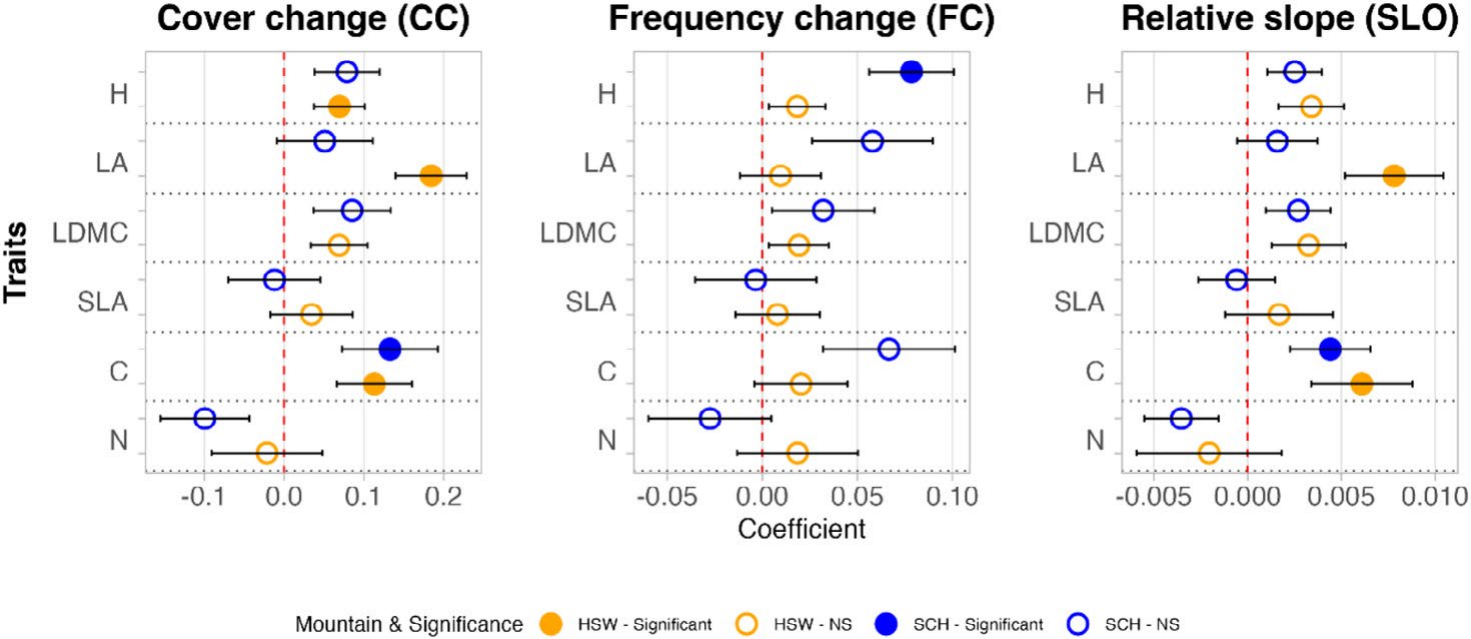
Relationships between functional traits and species abundance change at two mountain sites (Hochschwab (HSW) in orange, Schrankogel (SCH) in blue). Coefficients represent the change in relative cover change per scaled unit increase in each trait, based on linear models combining own and TRY trait data. Error bars show standard errors. Filled symbols indicate significant relationships (*p* < 0.05), open symbols non‐significant ones. Model details are provided in Table 4.

Incorporating TRY data revealed more statistically significant traits–change relationships at both sites (Figure 3, Table 4). At Hochschwab, leaf area, plant height, and carbon content were all significantly associated with cover change (LA: *R* ^2^ = 0.19, *p* < 0.001; H: *R* ^2^ = 0.04, *p* = 0.03; C: *R* ^2^ = 0.10, *p* = 0.02). Leaf area and leaf carbon content were also significant for relative slope (*R* ^2^ = 0.11, *p* = 0.004 and *R* ^2^ = 0.09, *p* = 0.03, respectively). At Schrankogel, significant relationships were fewer: leaf carbon content was associated with cover change and relative slope (CC: *R* ^2^ = 0.11, *p* = 0.03, SLO: *R* ^2^ = 0.10, *p* = 0.047), while height was significantly related to frequency change (*R* ^2^ = 0.17, *p* = 0.001).

**TABLE 4:** Summary of linear model performance for trait–abundance change relationships at two mountain sites (Hochschwab: HSW, Schrankogel: SCH), based on local and TRY trait data. *R* ^2^ and *p*‐values are shown for three measures of abundance change: Cover change (CC), frequency change (FC), and relative slope (SLO). N indicates the number of species per trait and site.

Trait	Mount	*N*_Obs	Cover change		Frequency change		Relative slope	
			*R* ^2^	*p*	*R* ^2^	*p*	*R* ^2^	*p*
H	HSW	126	0.037	0.031	0.012	0.218	0.03	0.053
	SCH	63	0.058	0.058	0.169	0.001	0.046	0.09
LA	HSW	75	0.19	0	0.003	0.656	0.109	0.004
	SCH	42	0.018	0.402	0.077	0.075	0.013	0.465
LDMC	HSW	91	0.041	0.055	0.017	0.225	0.03	0.102
	SCH	47	0.065	0.084	0.03	0.242	0.051	0.126
SLA	HSW	80	0.006	0.508	0.002	0.719	0.004	0.563
	SCH	48	0.001	0.836	0	0.913	0.002	0.776
C	HSW	55	0.098	0.02	0.013	0.411	0.088	0.028
	SCH	41	0.111	0.033	0.086	0.062	0.098	0.047
N	HSW	58	0.002	0.759	0.006	0.561	0.005	0.597
	SCH	42	0.073	0.083	0.018	0.399	0.074	0.082

## 4 Discussion

Alpine plant communities are undergoing rapid, well‐documented changes, including thermophilization (Lamprecht et al. 2018), shrub expansion (Lamprecht et al. 2021; Vanneste et al. 2026), and increasing species richness (Pauli et al. 2012; Steinbauer et al. 2018). At Schrankogel, this is reflected in the decline of cold‐temperature specialists, with notable losses of nival and subnival species even without obvious signs of competition (Steinbauer et al. 2020). We tested whether functional traits could help explain these changes. Contrary to our expectations, morphological (plant height and leaf area) and chemical traits (leaf carbon content), not physiological traits, tended to show more consistent associations with species responses, though overall explanatory power remained low (H1). Furthermore, functional traits showed similar response patterns across species response metrics (H2), suggesting that changes in cover, frequency, and slope are not governed by distinct physiological constraints.

The specific traits associated with species response differed between sites. At Hochschwab, plant height, leaf area, and leaf carbon content showed significant associations across multiple species response metrics, whereas at Schrankogel, only height and carbon content did, with leaf area showing no significant relationships. While trait—response patterns were broadly consistent across species response metrics, some differences emerged. Changes in frequency were associated with fewer traits, notably not carbon content, compared to cover change and slope. This is consistent with the expectation that cover change more closely reflects growth performance and resource acquisition, whereas frequency change captures short‐term establishment or colonization that does not necessarily indicate long‐term success (Helm et al. 2024; Lisner and Lepš 2020; Milberg et al. 2008). This interpretation is further supported by Rosbakh et al. (2025), who showed that seed and dispersal traits explained less than 20% of variation in alpine plant frequency change, a metric more sensitive to colonization and local extinction than cover (Madsen‐Hepp et al. 2023). Even traits directly linked to dispersal thus appear to have limited predictive power for frequency‐based responses.

In our study, morphological traits (height and leaf area) were significant predictors of species responses, consistent with previous studies demonstrating the importance of height (Bjorkman et al. 2018) and leaf morphological traits such as leaf thickness and SLA (Soudzilovskaia et al. 2013). Growing close to the ground is adaptive in low‐temperature alpine environments, where growth is restricted by temperature, and smaller plants benefit from the warmer microclimate near the soil surface (Körner 2021). As warming eases the low‐temperature constraints on growth and survival that historically filtered alpine plant communities, competitive interactions increasingly shape community assembly (Alexander et al. 2015; Körner 2021). Height thus becomes a key competitive trait in this context: experimental warming studies show that warming increases individual plant height and shifts community composition toward taller species, and community‐level height has risen across warming tundra and alpine sites (Bjorkman et al. 2018; Steinbauer et al. 2022; Quan et al. 2024). Because most alpine species are heliophilic (Landolt et al. 2010), they are particularly vulnerable to shading by taller competitors, and competition for light can drive competitive displacement more strongly than temperature constraints alone (Jaroszynska et al. 2024). At the same time, species may respond through different strategies—some by expanding leaf area to exploit increased resource availability, others by investing in structural height—with the latter potentially conferring a stronger long‐term competitive advantage despite its higher carbon cost (Craine and Dybzinski 2013). However, competitors can also weaken or modify trait selection pressures imposed by warming, primarily through suppressing fitness variation and altering microenvironmental conditions (Nomoto et al. 2024), making trait responses difficult to predict (García Criado et al. 2023).

While Soudzilovskaia et al. (2013) found SLA to be a significant predictor, our results showed leaf area rather than SLA as the significant leaf trait, suggesting absolute leaf size may be more important than leaf efficiency metrics in explaining species responses in our study system. The broader elevational range at Schrankogel, encompassing cold‐adapted nival and subnival species may also explain why leaf area was not a significant predictor at this site, as these high‐elevation specialists typically have smaller leaves adapted to extreme conditions (Körner 2021), potentially obscuring the competitive advantages associated with larger leaf area evident at the lower‐elevation Hochschwab site (Jaroszynska et al. 2024).

Species with high leaf carbon content increased in cover and the regression slope with time, but not in frequency. Leaf carbon content is frequently measured but is not part of the core trait sets commonly used to explain species responses to environmental change (Díaz et al. 2016; Wright et al. 2004). Carbon is a fundamental building block of organic matter, and leaf carbon concentration reflects the relative contributions of chemical compounds, such as lignin, rather than direct investment in growth. In our study, woody plants such as *Vaccinium* spp., *Empetrum nigrum, Salix* spp., and *Dryas octopetala* were among those with the highest leaf C content and generally increased in cover and frequency, indicating a “shrubification” also observed in the arctic, subarctic, and Mediterranean (Lamprecht et al. 2021; Maliniemi et al. 2025; Myers‐Smith et al. 2011; Vanneste et al. 2026). Plants with a high cell wall fraction—where lignin is found—tend to occupy the conservative end of the leaf economic spectrum, characterized by slow growth and resource conservation (Onoda et al. 2017; Wright et al. 2004). This contrasts with the expectation that a warmer alpine climate would favor faster‐growing, resource‐acquiring species, but conservative traits may confer unexpected advantages under changing environmental conditions (Soudzilovskaia et al. 2013).

The absence of frost and drought tolerance traits as significant predictors may reflect alternative strategies of alpine plants to cope with these stressors, or that these traits are not among those most relevant under selection from ongoing warming at these sites. Surviving low temperatures in alpine environments depends on multiple factors beyond intrinsic frost resistance, including snow cover protection and freezing avoidance mechanisms (Neuner 2014; Neuner et al. 2020; Taschler and Neuner 2004). We tested for summer leaf frost tolerance and expected the less tolerant species to increase as summer frosts declined (Gottfried et al. 2012), yet frost tolerance did not emerge as a significant predictor. Similarly, although we expected drought‐related traits to predict species responses, drought tolerance involves diverse strategies such as succulence, osmotic potential, and minimum conductance (Kramer 1983) that vary substantially with elevation and functional type (Rosbakh et al. 2017), and no single drought trait was significant. One temperature‐related stressor we did not measure is heat tolerance, and it may be the missing dimension most relevant to ongoing warming. For the most cold‐adapted high‐alpine specialists, heat stress may represent a fundamentally different challenge from the cold and drought stressors alpine plants have historically evolved under (Körner 2021). Species such as *Ranunculus glacialis* have the lowest heat tolerance and hardening capacity among alpine plants (Buchner and Neuner 2003), show reduced stress tolerance when grown under warm conditions (Streb et al. 2003), and exhibit photosynthetic collapse at temperatures that other alpine species can withstand (Larcher et al. 1997). Even acclimation to warmer growth temperatures does not confer resilience to heatwaves in such species (Notarnicola et al. 2021). Heat damage in plants is determined not by single temperature thresholds, but by the cumulative dose of heat exposure (temperature × duration) with prolonged heat waves reducing critical damage temperatures by 10°C–14°C compared to acute stress (Neuner and Buchner 2023). Moreover, high‐alpine specialists struggle to adjust dark respiration to higher temperatures (Larigauderie and Körner 1995), resulting in a negative carbon balance well below short‐term temperature thresholds and independent of competitive interactions. Together, these findings suggest that while alpine plants may tolerate brief temperature extremes, the extended warming periods characteristic of climate change could exceed their physiological capacity in ways not captured by the traits we measured.

Nevertheless, the traits we did examine revealed distinct patterns between our two study sites: trait–responses were broader at Hochschwab, with more traits showing significant relationships across species response metrics than at Schrankogel. These differences may partly reflect the contrasting geological substrates, community composition, elevational ranges, and number of species sampled. The geological substrates are ecologically significant determinants of species composition, with species richness responding differently to climate change on siliceous versus calcareous bedrock (Nicklas et al. 2021). Plots at Hochschwab span all four cardinal directions, whereas at Schrankogel they face south and east only. However, recent work in the European Alps suggests that topographic variation, including aspect, has limited influence on alpine plant community composition relative to the elevational gradient, which remains the dominant driver of both species distributions and community attributes (Chytrý et al. 2024). We therefore expect the difference in aspect coverage between our two sites to have had a minor influence on the observed trait–response patterns compared to the elevational and vegetation‐zone differences discussed below. The summits at Hochschwab are distributed over a relatively narrow elevation range (1910–2255 m) with calcareous bedrock, whereas Schrankogel spans a broader elevational gradient (2911–3456 m) with siliceous bedrock, including nival and sub‐nival zones, which are not found at Hochschwab. The nival and sub‐nival zones at Schrankogel harbor more specialized species, where harsh abiotic conditions constrain functional trait variability, while the lower‐elevation alpine communities at Hochschwab likely support a broader range of functional strategies (Körner 2021; López‐Angulo et al. 2020), potentially explaining the wider trait–response associations observed there. Moreover, vegetation changes tend to be more pronounced at lower elevations, declining toward higher and more extreme environments (Rumpf et al. 2018), and within Schrankogel itself, transformation was strongest at the lowest subsite (Lamprecht et al. 2018). The greater vegetation dynamics at Hochschwab's lower‐elevation summits may thus have provided a wider range of species responses for trait–abundance relationships to emerge, whereas the comparatively moderate changes in the nival and sub‐nival zones of Schrankogel may limit detectable trait–response patterns regardless of their ecological relevance.

Importantly, fewer significant correlations were found when only locally collected trait data were used. Incorporating TRY data substantially increased the number of species with trait values at both sites, with Hochschwab going from 48 to 143, and Schrankogel going from 33 to 73. The larger sample sizes likely contributed to the broader trait–response associations found in the combined dataset. While locally collected data offer the advantage of being measured at plants growing within the monitoring sites and using more uniform collection methods, both our local and combined datasets ignore intraspecific variation, which could influence the observed trait–change relationships (Albert 2015). Unfortunately, while including TRY data increases the number of species with morphological and chemical trait measurements, there were no additional species with physiological trait data. Moreover, while a significant association with leaf area at Hochschwab did emerge from the locally measured data alone, most trait–abundance associations were significant only when additional data from TRY were included. This reflects the increase in statistical power from larger sample sizes, and means that the *p*‐values alone should be interpreted with some caution: the strength of the signal, as much as the underlying ecological relationships, depends on data availability.

A further caveat concerns the overall explanatory power of our models. Even where trait–abundance associations were statistically significant, *R* ^2^ values remained below 0.20, indicating that the functional traits measured can explain only a small‐to‐moderate share of the observed changes in species abundance. Additional unmeasured factors—biotic interactions such as competition and facilitation, and site‐specific processes such as snow cover dynamics and grazing history—are likely to contribute substantially to the observed patterns and should be considered in future trait‐based studies of alpine vegetation change.

Overall, measured morphological traits, particularly plant height and leaf area, were more reliable predictors of changes in alpine species abundance than physiological traits, contradicting our hypothesis that physiological tolerance would be most relevant under climate change. This points to indirect effects of warming, mediated through competitive interactions, as a stronger driver of community change than direct physiological stress, while the significance of leaf carbon content suggests conservative strategies may also be advantageous for some species. Future research should target physiological traits not captured here, particularly heat tolerance, and examine how the balance between competition and physiological stress shifts under continued warming.

## Author Contributions

**Feline Peters:** conceptualization (supporting), data curation (equal), formal analysis (lead), investigation (supporting), methodology (supporting), software (lead), validation (equal), visualization (lead), writing – original draft (lead), writing – review and editing (lead). **Jana Weghorst:** data curation (equal), investigation (supporting). **Mariana Paetzolt:** data curation (equal), investigation (supporting). **Andrea Lamprecht:** data curation (equal), investigation (supporting), writing – review and editing (supporting). **Harald Pauli:** data curation (equal), funding acquisition (lead), investigation (lead), project administration (lead), resources (lead), writing – review and editing (supporting). **Manuela Winkler:** data curation (equal), investigation (supporting), writing – review and editing (supporting). **Patrick Saccone:** data curation (equal), investigation (supporting), writing – review and editing (supporting). **Peter Hietz:** conceptualization (lead), data curation (equal), formal analysis (supporting), funding acquisition (lead), investigation (lead), methodology (lead), project administration (lead), resources (lead), software (supporting), supervision (lead), validation (equal), visualization (supporting), writing – original draft (supporting), writing – review and editing (supporting).

## Funding

GLORIA vegetation survey in 2015 was funded by the Austrian Academy of Sciences (project MEDIALPS—Disentangling anthropogenic drivers of climate change impacts on alpine plant species: Alps vs. Mediterranean mountains), and the surveys in 2022 and 2023 were funded by the European Research Council (ERC) under the European Union's Horizon 2020 research and innovation programme (MICROCLIM, grant agreement no. 883669). The Government of Tyrol provided permission to establish a field camp at Schrankogel in 2023.

## Conflicts of Interest

The authors declare no conflicts of interest.

## Supporting information


**Table S1:** Coverage of trait data used in the linear models, given as the number of species with available values per trait at each study site. The ‘local’ columns refer to species for which traits were measured locally; the ‘combined’ columns include species supplemented with values from the TRY plant trait database (Kattge et al. 2020). Traits are grouped by category (morphological, physiological, chemical, and the first two principal components from a PCA on morphological traits). Traits are grouped by category (morphological, physiological, chemical, and the first two principal components from a PCA on morphological traits). The ‘local’ columns refer to species for which trait values were measured locally; the ‘combined’ columns include species supplemented with values from the TRY plant trait database (Kattge et al. 2020) to broaden species coverage. Trait abbreviations: H = plant height, LA = leaf area, SLA = specific leaf area, LDMC = leaf dry matter content, LT = leaf thickness, AR = aspect ratio, Solidity = leaf solidity, gmin = minimum leaf conductance, Osm = osmotic potential, Chl = chlorophyll content, Suc = succulence, LT_50_ = lethal temperature (frost tolerance), C = leaf carbon content, *N* = leaf nitrogen content. Site abbreviations: SCH = Schrankogel, HSW = Hochschwab.
**Table S2:** Species loadings on the first two principal components (PC1 and PC2) from PCA analysis of Schrankogel (SCH) data.
**Table S3:** Species loadings on the first two principal components (PC1 and PC2) from PCA analysis of Hochschwab (HSW) data.
**Table S4:** Trait loadings on the first two principal components (PC1 and PC2) from PCA analysis of Schrankogel (SCH) & Hochschwab (HSW) data.

## Data Availability

All data and code supporting this study are provided as Supporting Information, including the species abundance change data, functional trait measurements, imputed trait data, extracts from the TRY Plant Trait Database (Kattge et al. 2020), and the R project with R Markdown analysis scripts. TRY data were obtained under request number 21197; access to the full TRY database requires submission of a data request via https://www.try‐db.org.
