# Supplementary material for: Can Functional Traits Explain Recent Changes in Abundance of Alpine Plant Species?
Source: Ecol Evol. 2026 Jun 7;16(6):e73806. doi: 10.1002/ece3.73806 (PMC13243881; doi:10.1002/ece3.73806)
Supplement: Supplementary file 1 — Table S1: Coverage of trait data used in the linear models, given as the number of species with available values per trait at each study site. The ‘local’ columns refer to species for which traits were measured locally; the ‘combined’ columns include species supplemented with values from the TRY plant trait database (Kattge et al. 2020). Traits are grouped by category (morphological, physiological, chemical, and the first two principal components from a PCA on morphological traits). Traits are grouped by category (morphological, physiological, chemical, and the first two principal components from a PCA on morphological traits). The ‘local’ columns refer to species for which trait values were measured locally; the ‘combined’ columns include species supplemented with values from the TRY plant trait database (Kattge et al. 2020) to broaden species coverage. Trait abbreviations: H = plant height, LA = leaf area, SLA = specific leaf area, LDMC = leaf dry matter content, LT = leaf thickness, AR = aspect ratio, Solidity = leaf solidity, gmin = minimum leaf conductance, Osm = osmotic potential, Chl = chlorophyll content, Suc = succulence, LT50 = lethal temperature (frost tolerance), C = leaf carbon content, N = leaf nitrogen content. Site abbreviations: SCH = Schrankogel, HSW = Hochschwab. Table S2: Species loadings on the first two principal components (PC1 and PC2) from PCA analysis of Schrankogel (SCH) data. Table S3: Species loadings on the first two principal components (PC1 and PC2) from PCA analysis of Hochschwab (HSW) data. Table S4: Trait loadings on the first two principal components (PC1 and PC2) from PCA analysis of Schrankogel (SCH) & Hochschwab (HSW) data. [file ECE3-16-e73806-s001.docx]

# Supplementary information

## Our field measurement protocols

Leaves were collected from individual plants (five leaves per species) and processed in the laboratory. For each plant, one leaf (excluding the petiole) was scanned using a portable scanner (HP Scanjet G31110 Photo Scanner) at 300 dpi resolution. Leaf area, aspect ratio (leaf length/leaf width), and solidity (area/convex hull area) were calculated using ImageJ software (imagej.nih.gov). Chlorophyll content was measured at three positions on each leaf using a SPAD-502Plus Chlorophyll Meter, and the average SPAD value was recorded. SPAD units are proportional to chlorophyll content (Ling, et al., 2011). Leaf thickness was measured with a Helios thickness gauge (accuracy: 0.01 mm). Leaves were saturated overnight by wrapping them in wet tissue paper and sealing them in Ziplock bags. The next day, leaves were dark-adapted for 20 minutes, blotted dry, and weighed to the nearest 0.1 mg. Chlorophyll fluorescence (Fv/Fm) was measured using a Mini-PAM Photosynthesis Yield Analyzer (Walz GmbH, Effeltrich, DE). The Fv/Fm ratio, calculated as variable fluorescence (Fv) divided by maximum fluorescence (Fm), indicates photosynthetic efficiency, with a value of c. 0.83 representing a non-stressed leaf (Jägerbrand & Kudo, 2016). For the minimum conductance, initial weight and Fv/Fm were recorded before leaves were placed on a wire mesh in front of two small fans under low light (< 2 µmol m(^{-2}) s(^{-1})). Leaves were identified using color-coded clips, which were weighed and attached to each sample. Weight and Fv/Fm were measured over 2–3 days, with measurement intervals starting at ~15 minutes and increasing as water loss slowed. Temperature (T) and relative humidity (rH) were recorded using data loggers, and vapor pressure deficit (VPD) was calculated as:

$$Vapor pressure deficit= \frac{\left( \frac{1-rH}{100} \right)*0.6107*{10}^{7.5*T}}{(237.3+T)}$$

Where rH is the relative humidity (%) and T is the temperature in ℃. Since VPD drives evaporation and was not constant during the measurements, it is needed to calculate minimum conductance (Sack & Scoffoni, 2010). Using the weight loss data, the relative water content (RWC) was then calculated as:

$$RWC= \frac{fresh weight-dry weight}{saturated weight-dry weight}$$

The RWC was then used to fit the following function from Cape and Percy (1996) to the data from the leaf drying curves:

$$RWC_{t}\sim R_{e}+\left( R_{0}-R_{e} \right)*exp^{-kt^{'}}$$

The constant k was then used to calculate g_min,_ which is the minimal epidermal conductance. When stomata are closed, this is water loss through leaky stomata and the cuticle, the boundary layer being strongly reduced (Cape & Percy, 1996) the fan. It was calculated using Suc (water per leaf area), with the following equations:

$$Suc= \frac{(sat fresh weight -dry weight )}{LA}$$

$$g_{min}=Succulence*k$$

After the weight-loss measurements, the leaves were dried in a drying oven at 80 °C for at least 72 hrs and then weighed to measure the dry weight. The saturated fresh weight was measured as the max. fresh weight. Using those measurements, the leaf dry matter content (LDMC) was calculated as:

$$LDMC= \frac{dry weight}{sat. fresh weight}$$

Using the LA and the dry weight, the SLA (ratio leaf area to leaf weight) was calculated:

$$SLA= \frac{LA}{dry weight}$$

Additional leaves were cut into small pieces and placed in Eppendorf vials (0.8 mL) with filter inlets (0.2 mL). Samples were frozen at -20 °C in the field and transported to Vienna, where they underwent freeze-thaw cycles to break cell membranes. Vials were centrifuged at 25 °C (14,000 rpm, 20,160 g) for 10 minutes, and the osmotic concentration of the extracted liquid was measured using a vapor pressure osmometer (VAPRO®, model 5520). Forty additional leaves per species were collected, mixed, and placed on transparent plastic sheets (1–10 leaves per circle, depending on size). Leaves were attached with Transpore™ tape, wrapped in moist tissue paper, and treated with an ice nucleation agent (Snomax® suspension) to prevent supercooling. Samples were sealed in plastic bags and submerged in a 50:50 water/ethylene glycol mixture. The temperature was lowered at a rate of 4 °C/h, held at target temperatures (+4, -4, -6, -9.5, -11.5, -14, -16.5, -17 °C) for 2 hours, and then warmed back to 4 °C at the same rate. Samples kept at +4 °C served as controls. After exposure, leaves were dark-adapted for 20 minutes and Fv/Fm was measured using a GFS-3000 (Walz, Effeltrich). The temperature at which Fv/Fm declined by 50% (LT50) was determined by fitting a sigmoidal function to the frost resistance curve.

**Refereces:**

1. Cape, J. N., & Percy, K. E. (1996). The interpretation of leaf‐drying curves. Plant, Cell & Environment, 19(3), 356–361. https://doi.org/10.1111/j.1365-3040.1996.tb00258.x
2. Jägerbrand, A., & Kudo, G. (2016). Short-Term Responses in Maximum Quantum Yield of PSII (Fv/Fm) to ex situ Temperature Treatment of Populations of Bryophytes Originating from Different Sites in Hokkaido, Northern Japan. *Plants*, *5*(2), 22. <https://doi.org/10.3390/plants5020022>
3. Ling, Q., Huang, W., & Jarvis, P. (2011). Use of a SPAD-502 meter to measure leaf chlorophyll concentration in Arabidopsis thaliana. Photosynthesis Research, 107(2), 209–214. https://doi.org/10.1007/s11120-010-9606-0
4. Sack, L., & Scoffoni, C. (2010). Minimum epidermal conductance (gmin, a.k.a. Cuticular conductance). PrometheusWiki. https://prometheusprotocols.net/function/gas-exchange-and-chlorophyll-fluorescence/stomatal-and-non-stomatal-conductance-and-transpiration/minimum-epidermal-conductance-gmin-a-k-a-cuticular-conductance/

## Supplementary information - Tables

Table S1: Coverage of trait data used in the linear models, given as the number of species with available values per trait at each study site. The 'local' columns refer to species for which traits were measured locally; the 'combined' columns include species supplemented with values from the TRY plant trait database (Kattge et al., 2020). Traits are grouped by category (morphological, physiological, chemical, and the first two principal components from a PCA on morphological traits).. Traits are grouped by category (morphological, physiological, chemical, and the first two principal components from a PCA on morphological traits). The 'local' columns refer to species for which trait values were measured locally; the 'combined' columns include species supplemented with values from the TRY plant trait database (Kattge et al., 2020) to broaden species coverage. Trait abbreviations: H = plant height, LA = leaf area, SLA = specific leaf area, LDMC = leaf dry matter content, LT = leaf thickness, AR = aspect ratio, Solidity = leaf solidity, gmin = minimum leaf conductance, Osm = osmotic potential, Chl = chlorophyll content, Suc = succulence, LT₅₀ = lethal temperature (frost tolerance), C = leaf carbon content, N = leaf nitrogen content. Site abbreviations: SCH = Schrankogel, HSW = Hochschwab.

| **Category** | **Trait** | **SCH_local** | **SCH_combined** | **HSW_local** | **HSW_combined** |
| --- | --- | --- | --- | --- | --- |
| *Morphological* | *H* | 33 | 63 | 39 | 126 |
| *Morphological* | *LA* | 33 | 42 | 39 | 75 |
| *Morphological* | *SLA* | 33 | 48 | 39 | 80 |
| *Morphological* | *LDMC* | 33 | 47 | 39 | 91 |
| *Morphological* | *LT* | 29 | NA | 38 | NA |
| *Morphological* | *AR* | 33 | NA | 39 | NA |
| *Morphological* | *Solidity* | 24 | NA | 39 | NA |
| *Physiological* | *gmin* | 23 | NA | 31 | NA |
| *Physiological* | *Osm* | 32 | NA | 33 | NA |
| *Physiological* | *Chl* | 20 | NA | 33 | NA |
| *Physiological* | *Suc* | 33 | NA | 39 | NA |
| *Physiological* | *LT_50* | 28 | NA | 36 | NA |
| *Chemical* | *C* | NA | 41 | NA | 55 |
| *Chemical* | *N* | NA | 42 | NA | 58 |
| *PCA* | *PC1* | 33 | NA | 44 | NA |
| *PCA* | *PC2* | 33 | NA | 44 | NA |

*Table S2: Species loadings on the first two principal components (PC1 and PC2) from PCA analysis of Schrankogel (SCH) data.*

| **Species** | **PC1** | **PC2** |
| --- | --- | --- |
| *Agrostis rupestris* | -4,1341 | 3,187108 |
| *Androsace alpina* | 0,509972 | 1,107183 |
| *Arenaria ciliata* | 1,681984 | 1,963673 |
| *Avenula versicolor* | -4,20892 | -0,87097 |
| *Campanula scheuchzeri* | -1,86429 | -0,30706 |
| *Carex curvula* | -3,03471 | -3,05297 |
| *Cerastium uniflorum* | 1,812231 | 0,709014 |
| *Erigeron uniflorus* | 1,395994 | 0,251352 |
| *Gentiana bavarica* | 0,527455 | 0,655811 |
| *Geum montanum* | -0,55039 | -2,52923 |
| *Geum reptans* | 0,132256 | -2,2105 |
| *Homogyne alpina* | 1,318894 | -2,25584 |
| *Leucanthemopsis alpina* | 0,036721 | -0,8963 |
| *Linaria alpina* | 0,902452 | 0,543543 |
| *Luzula spicata* | -1,66627 | -0,00696 |
| *Minuartia sedoides* | -0,37767 | 2,719006 |
| *Minuartia verna* | -0,81555 | 2,779885 |
| *Oreochloa disticha* | -3,31119 | -1,24819 |
| *Pedicularis aspleniifolia* | 1,394605 | -1,27522 |
| *Phyteuma hemisphaericum* | -2,58536 | 0,176528 |
| *Poa alpina* | -3,53266 | 0,22412 |
| *Poa laxa* | -2,14961 | 1,156661 |
| *Polygonum viviparum* | 0,368116 | -1,49546 |
| *Potentilla aurea* | -0,36892 | -0,11893 |
| *Primula glutinosa* | 2,133084 | -1,45198 |
| *Primula hirsuta* | 1,6542 | -0,78433 |
| *Primula minima* | 2,044451 | 0,121047 |
| *Ranunculus glacialis* | 1,379566 | -3,3279 |
| *Salix herbacea* | 0,00983 | 0,240649 |
| *Saxifraga bryoides* | 2,018023 | 1,803015 |
| *Saxifraga exarata* | 1,055272 | 0,735108 |
| *Saxifraga oppositifolia* | 1,30669 | 1,797302 |
| *Sedum alpestre* | 2,822002 | -0,09751 |
| *Sibbaldia procumbens* | -0,44445 | 0,641248 |
| *Silene acaulis* | 1,162631 | 1,978904 |
| *Thymus praecox* | 1,729991 | 1,296854 |
| *Veronica alpina* | 1,004074 | -0,58654 |
| *Veronica bellidioides* | 0,643605 | -1,57211 |

*Table S3: Species loadings on the first two principal components (PC1 and PC2) from PCA analysis of Hochschwab (HSW) data.*

| **Species** | **PC1** | **PC2** |
| --- | --- | --- |
| *Agrostis rupestris* | -2,96323 | -2,17081 |
| *Androsace chamaejasme* | 2,817754 | 0,178697 |
| *Arenaria ciliata* | 1,780038 | -3,49903 |
| *Aster bellidiastrum* | 0,716646 | 0,324455 |
| *Bartsia alpina* | -0,0171 | -0,20294 |
| *Campanula alpina* | 0,807698 | -2,37251 |
| *Campanula scheuchzeri* | -1,90461 | -1,03272 |
| *Carex atrata* | -3,44401 | 1,008629 |
| *Carex firma* | -2,15247 | 0,420902 |
| *Carex sempervirens* | -4,13734 | 0,111418 |
| *Crepis aurea* | 0,446503 | -1,94271 |
| *Crepis terglouensis* | 2,921363 | 0,857982 |
| *Dianthus alpinus* | 0,963314 | -1,67639 |
| *Doronicum glaciale* | 2,150862 | 1,76388 |
| *Dryas octopetala* | 0,543044 | 1,322165 |
| *Erigeron uniflorus* | 1,139743 | 0,644623 |
| *Festuca versicolor* | -3,97328 | 1,201834 |
| *Gentiana clusii* | 0,71412 | 2,28573 |
| *Geranium sylvaticum* | -0,42161 | 0,598945 |
| *Geum montanum* | -0,31863 | 2,29639 |
| *Helianthemum nummularium* | 1,059279 | -0,243 |
| *Helianthemum oelandicum* | 0,756686 | -0,07865 |
| *Homogyne alpina* | 0,973469 | 1,562563 |
| *Homogyne discolor* | 0,616473 | 4,092384 |
| *Juncus trifidus* | -2,37344 | 0,625123 |
| *Kobresia simpliciuscula* | -4,1492 | -0,99437 |
| *Leontodon hispidus* | 0,687717 | -0,99867 |
| *Luzula glabrata* | -3,4921 | 1,547403 |
| *Minuartia sedoides* | -0,30665 | -0,80578 |
| *Minuartia verna* | -0,61983 | -3,23191 |
| *Pedicularis rosea* | 1,35278 | -0,60949 |
| *Petrocallis pyrenaica* | 2,797587 | 0,81761 |
| *Poa alpina* | -1,94417 | -1,40141 |
| *Polygonum viviparum* | 0,800246 | 0,390531 |
| *Potentilla aurea* | -0,39283 | 0,521882 |
| *Potentilla clusiana* | -0,14308 | -0,63179 |
| *Primula auricula* | 2,538676 | 1,825211 |
| *Primula clusiana* | 0,479424 | 0,34271 |
| *Ranunculus alpestris* | 0,913634 | 0,69087 |
| *Ranunculus montanus* | 0,910152 | -0,33983 |
| *Salix reticulata* | 0,213264 | 1,667304 |
| *Salix retusa* | 0,931837 | -0,06279 |
| *Saxifraga aizoides* | 1,964769 | -0,67182 |
| *Saxifraga exarata* | 0,675857 | -1,48769 |
| *Saxifraga paniculata* | 2,599452 | 1,97006 |
| *Sesleria albicans* | -4,13303 | 0,591863 |
| *Silene acaulis* | 2,017078 | -2,85321 |
| *Solidago virgaurea* | -0,62145 | 0,026511 |
| *Thymus praecox* | 1,309084 | -1,61516 |
| *Trifolium badium* | -0,54788 | 0,255288 |
| *Vaccinium myrtillus* | -1,54669 | -0,58236 |
| *Vaccinium vitis-idaea* | -0,68597 | 1,652906 |
| *Valeriana celtica* | 0,889893 | -0,51256 |
| *Viola biflora* | 0,800156 | -1,57826 |

*Table S4: Trait loadings on the first two principal components (PC1 and PC2) from PCA analysis of Schrankogel (SCH) & Hochschwab (HSW) data.*

|  | HSW | | SCH | |
| --- | --- | --- | --- | --- |
| **variable** | **PC1** | **PC2** | **PC1** | **PC2** |
| *AR* | -0,36925 | -0,12518 | -0,41253 | 0,040027 |
| *Chl* | 0,117078 | 0,516168 | 0,258601 | -0,28094 |
| *gmin* | 0,126422 | -0,11526 | -0,14493 | 0,317346 |
| *H* | -0,33659 | 0,112751 | -0,2906 | -0,36492 |
| *LA* | -0,09703 | 0,357721 | -0,11671 | -0,50427 |
| *LDMC* | -0,3648 | 0,262585 | -0,3188 | 0,040348 |
| *LT* | 0,227161 | 0,444662 | 0,123643 | -0,47403 |
| *LT_50* | 0,384193 | -0,17169 | 0,364971 | 0,174812 |
| *Osm* | -0,35441 | 0,256717 | -0,39338 | -0,27207 |
| *SLA* | -0,03082 | -0,37941 | -0,04419 | 0,062009 |
| *Solidity* | 0,307581 | 0,160458 | 0,352113 | -0,06969 |
| *Suc* | 0,397245 | 0,179383 | 0,336243 | -0,3028 |
